# The potential role of miR-27a and miR-320a in metabolic syndrome in obese Egyptian females

**DOI:** 10.1186/s43141-022-00348-x

**Published:** 2022-05-19

**Authors:** Amira Mohamed Abd El-Jawad, Iman Hassan Ibrahim, Moushira Erfan Zaki, Tahany Ramzy Elias, Wafaa Ibrahim Rasheed, Khalda Said Amr

**Affiliations:** 1 Department of Medical Biochemistry, National Research Centre, Cairo, Egypt; 2 Department of Biochemistry, Faculty of Pharmacy for Girls, Al-Azhar University, Cairo, Egypt; 3 Department of Biological Anthropology, National Research Centre, Cairo, Egypt; 4 Department of Medical Molecular Genetics, National Research Centre, Cairo, Egypt

**Keywords:** Metabolic syndrome, Obesity, Hyperglycemia, Hyperlipidemia, miR-27a, miR-320a, Insulin resistance, Case-control observational study

## Abstract

**Background:**

Metabolic syndrome (MetS) is a combination of many health complications, such as obesity, high blood pressure, hyperlipidemia, hyperglycemia, and insulin resistance, with an increasing threat of type 2 diabetes mellitus (T2DM) and cardiovascular diseases. As the MetS develops, an alteration in the expression of some genes regulated by circulating microRNAs may also develop as a consequence. TaqMan microRNA primers specific for both miR-27a and miR-320a were used to estimate their expression levels in plasma samples collected from two groups: obese females with metabolic syndrome (*n* = 49) and lean healthy female volunteers (*n* = 23), to detect if their expression levels were deregulated with MetS.

**Results:**

The study results revealed that miR-27a was upregulated in the plasma of MetS group compared to the healthy controls, while miR-320a was downregulated (*p* ≤ 0.005). There was a highly significantly positive correlation between miR-27a expression and body mass index (BMI), waist circumference (WC), fasting blood glucose (FBG), insulin resistance (represented as HOMA-IR), and triglycerides (TG), while it showed significantly negative correlation only with HDL-cholesterol (*p* ≤ 0.0001). miR-320a showed significantly negative correlation with BMI, WC, waist-hip ratio (WHR), FBG, HOMA-IR, and TG. The expression value of miR-320a was positively correlated with HDL-cholesterol. Area under the curves (AUC) was equal to 1.000 for both microRNAs.

**Conclusion:**

Our study added more evidence that monitoring changes in expression levels of both miR-27a and miR-320a in MetS patients could help in the evaluation of disease progression, risk, and susceptibility.

## Background

Metabolic syndrome (MetS) is a critical global health issue which embraces a group of different metabolic disorders such as hyperglycemia, dyslipidemia, visceral obesity, insulin resistance (IR), nonalcoholic fatty liver (NAFL), and hypertension [21] resulting in an increased risk of type 2 diabetes mellitus (T2DM), cardiovascular disease (CVD), fatty liver, and tumors [26]. The National Cholesterol Education Programs Adult Treatment Panel III (NCEP ATP III) presented a simple set of diagnostic criteria for MetS based on waist circumference (WC), fasting blood glucose (FBG), triglycerides (TG), high-density lipoprotein-cholesterol (HDL-C) and blood pressure (BP). In 2005, the International Diabetes Federation (IDF) adjusted the MetS definition, which stated that waist circumference is essential for the MetS diagnosis along with two or more of the MetS variables [28].

Metabolic syndrome is though being high in both sexes, but the pooled pervasiveness of MetS seems to be higher in females than in males [14]. The search for improved and novel prognostic strategies forced the research area to revert back to illuminating the molecular mechanisms underlying the MetS [12].

Not long ago, a certain type of cell-free, noncoding RNAs, named microRNAs, is appearing as key regulators of metabolism [19]. Those noncoding RNAs (ncRNAs) are produced from the bigger part of the genome that does not code for proteins, but bring about noncoding transcripts that modulate protein function and gene expression [29, 33] by joining to 3′-untranslated region (3′-UTR) in their target messenger RNA (mRNA) [8]. This regulation alters the protein translation process either by repression or degradation [8].

There is a growing proof assisting the function of extra- and intracellular microRNAs as contributing factors of the exchange between liver, adipose tissues, skeletal muscle, and other organs, targeting the paracrine connection between those distinct tissues [19]. Moreover, the expression profile of microRNAs was found to be altered in tissue samples and in plasma with some diseases like hyperlipidemia, tumors, endothelial cell dysfunction, hyperglycemia, and diabetes [6]. MicroRNAs correlation to the disease severity and their release into the circulation became the center of attention not only as hopeful noninvasive circulating diagnostic and prognostic markers [7, 25] but also as therapeutic targets that might represent a very promising approach for the treatment of many diseases [33].

The miR-27a is one of the most critical and important microRNAs recognized until now. This is because of its role in the regulation of many biological processes. It has been clarified that miR-27a might play a pivotal part in the insulin signaling pathways related to glucose metabolism and IR [5].

By studying the expression profile of microRNAs in disorders connected to lipid and glucose metabolism, scientists have found that miR-320a could be a possible biomarker [6]. There are some reports recommending that it plays various roles in metabolism-associated injuries like provoking diabetes, cardiac dysfunction [16], increasing the risk of atherosclerosis [4], and deteriorating diabetic nephropathy [13]. Other reports recommend that it may play a probable role in the therapy of chronic diabetic complications, as it is able to regulate many transcripts [11].

Our study aimed to characterize the relative expression of these microRNAs and question their relationship with the different metabolic variables in a group of Egyptian adult females with and without metabolic syndrome, which might reveal novel clinical and therapeutic limits for diabetes avoidance and therapy, obesity, and related metabolic complications.

## Subjects and methods

### Patients

A total of 72 adult females were recruited from the obesity and internal medicine clinics of the National Research Center (NRC). The study was approved by Ethical Committee on Human Research in the National Research Center. Enrolled subjects were informed about the aims, methods, and possible results of the study, and all gave their consent. No participants are under 18. The patients were classified into two groups according to their BMI and MetS diagnosis: (1) control group with a BMI < 30 and no clinical diagnosis of MetS and (2) MetS patients with their diagnosis based on the National Cholesterol Education Program Adult Treatment Panel III (NCEP ATP III) definition. Patients with malignancy or those with age range less than 18 or more than 45 years old were excluded from this study. Weight, height, waist circumference (WC) (midway from the lowest rib to the iliac crest to the nearest 0.1 cm), and blood pressure of each patient were measured. BMI was calculated for each participant by dividing weight (kg) by the square of height (m).

### Blood samples

Blood samples were drawn from an antecubital vein in EDTA tubes [25] after 8 h fasting, to examine levels of microRNAs, blood glucose, lipid profiles, and insulin levels. Blood samples were first treated by centrifugation at 4500 rpm for 10 min at 4 °C. Then plasma samples were kept at −40 °C in aliquots until processed. Hemolyzed plasma samples were excluded [18].

### Expression of microRNA 27a and microRNA 320a by RNA extraction and RT-quantitative PCR

RNeasy Mini Kit (QIAGEN, Hilden, Germany) was used for RNA isolation according to the manufacturer’s directions for purification of miRNA, and then the isolated RNA was stored at −80 °C. MicroRNA expression for miR-27a and miR-320a were set on by applying the TaqMan MicroRNA Assays (Applied Biosystems, Carlsbad, CA, USA). Reverse Transcription Kit (Applied Biosystems) was used to further reverse-transcribe the extracted microRNA; this step took place in the reaction mixture containing miR-specific stem-loop RT primers for each. Master Mix of TaqMan Universal PCR without AmpErase UNG (Applied Biosystems) was adjusted for real-time PCR, ABI PRISM 7000 system (Applied Biosystems). The provoked microRNA data were calculated relative to a RUN6B. All samples were measured in duplicates, and relative quantity (Rq) of miR-27a and miR-320a was calculated by the formula (*Rq* = 2^−ΔΔCt^).

### Statistical analysis

Analyses of data was done using Statistical Package for the Social Sciences (SPSS) software (SPSS Inc., Chicago, USA), version 19.0. Results are expressed as mean ± standard deviation (SD). At *p* ≤ 0.05, statistical differences were considered significant. In order to check normality, the Shapiro-Wilk test was used. Independent sample Student’s *t*-test, with one-tailed alternative hypothesis was run to determine the differences in means of the studied variables between the studied groups. MicroRNA expression levels were compared using Mann-Whitney’s *U*-test. Spearman’s rank correlation coefficient (*r*) was calculated, and testing the significance of *r* was done to determine if there is correlation between metabolic syndrome parameters and the expression of each microRNA. Data were further supported by receiver operating characteristic (ROC) analyses, and AUCs were calculated to evaluate the sensitivity and specificity of all analyses. The best cutoff is defined by Youden index as the point that has the highest sum of sensitivity and specificity [10, 21].

## Results

In the clinic, 72 female individuals were enrolled into our study and placed in one of two groups: females with a normal BMI and no MetS (*n* = 23) and obese female participants with a clinical diagnosis of metabolic syndrome (*n* = 49). The mean age ratio showed no difference between the two groups. Participants with metabolic syndrome showed significant differences in all of the clinical parameters compared to those without metabolic syndrome except for the blood pressure.

According to NCEP ATP III criteria, BMI, FBG, and insulin resistance (HOMA-IR) were significantly higher among metabolic syndrome group compared to control group at *p* ≤ 0.0005; also, there was a significant difference in WC and serum insulin between patients with MetS and controls at *p* ≤ 0.005 for the former and *p* ≤ 0.05 for the latter. Regarding lipid profile parameters, the mean serum level of total cholesterol, TG, LDL-cholesterol, and VLDL-cholesterol was significantly higher in MetS group as compared to controls, while the mean serum level of HDL-cholesterol proved a significant decrease in MetS group compared to controls (all at *p* ≤ 0.0005). Results are shown in Table 1.

**Table 1:** Mean ± SD of different parameters in the studied groups

Parameters	***Groups***		***p-value***
	Group 1 (control ***n*** = 23)	Group 2 (metabolic syndrome ***n*** = 50)	
**Body mass index (BMI), kg/m**^**2**^	23.6073 ± 2.874	33.59 ± 6.7092***	***P*** **= 0.0001**
**Waist circumference (cm)**	79.3043 ± 5.506	112.08 ± 11.11**	***P*** **= 0.0041**
**Fasting blood glucose, mg/dl**	80.21 ± 12.44	193.6 ± 50.2***	***P*** **= 0.0001**
**Serum insulin, μ IU/ml**	11.8457 ± 3	16.652 ± 3.9*	***P*** **= 0.013**
**Insulin resistance, HOMA-IR**	2.3207 ± 0.9	8.25 ± 2***	***P*** **= 0.0001**
**Total cholesterol, mg/dl**	159.4783 ± 26.1688	197 ± 44.49811***	***P*** **= 0.0001**
**Triglycerides, mg/dl**	60.3043 ± 22.85216	177.32 ± 30.21***	***P*** **= 0.0001**
**LDL-cholesterol, mg/dl**	79.5217 ± 25.41385	122.436 ± 22.12279***	***P*** **= 0.0001**
**VLDL-cholesterol, mg/dl**	12.0609 ± 4.57043	35.464 ± 13.78567***	***P*** **= 0.0001**
**HDL-cholesterol, mg/dl**	79.5217 ± 18.76377	41.48 ± 9.37633***	***P*** **= 0.0001**

*Means significant with *P* < 0.05. **Means significant with *P* < 0.005. ***Means significant with *P* < 0.0001

### Circulating miRNA biomarkers

**Fig. 1:**
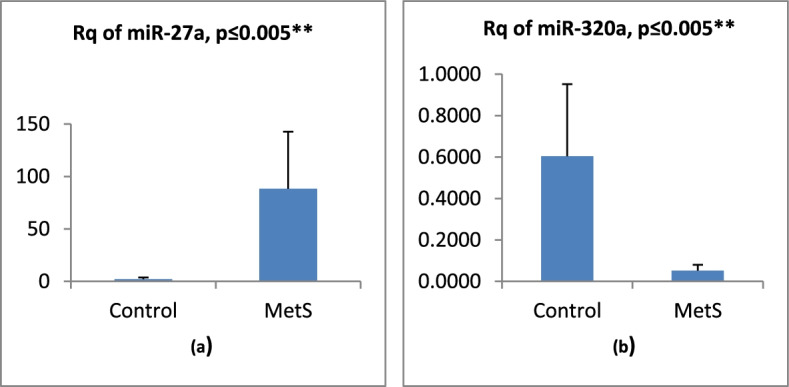
**a**, **b** Mean (SD) of miR-27a and miR-320a relative quantification, where “**” means significant at *P* < 0.005

On comparing the relative expression (Rq) levels of miR-27a, it showed a significant and dramatic increase in the patients compared to controls, while miR-320a expression level revealed a significant decrease compared to control group as shown in Fig. 1.

**Fig. 2:**
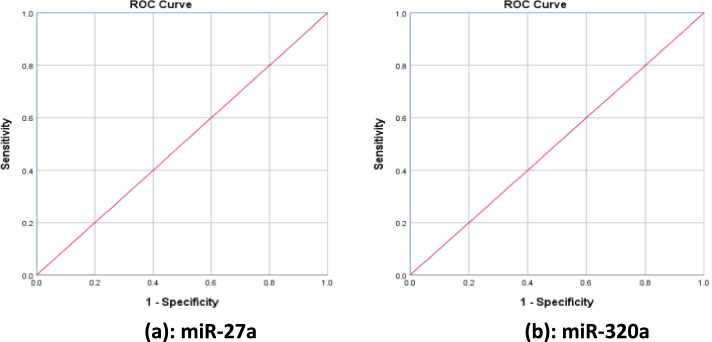
**a**, **b** ROC curve of Rq of miR-27a (*AUC* = 1.000, cutoff ≥ 13.61) and miR-320a (*AUC* = 1.000, cutoff ≤ 0.122) in discriminating control group from metabolic syndrome group

The (ROC) curves of all laboratory tests were constructed and statistically analyzed for both studied microRNAs Rq analysis to calculate their best cutoff points. As for miR-27a, the best cutoff point to discriminate the control group from the MetS group is ≥ 13.61 at absolute sensitivity (true positive) and absolute specificity (true negative). Area under the curve (AUC) [SE] = 1.000, *p* = 0.0001 (Table 2, Fig. 2). While the ROC curve analysis for miR-320a relative expression analysis showed that the best cutoff point is ≤ 0.122 at which sensitivity = 100 and specificity = 100, area under the curve (AUC) [SE] = 1 at *p* = 0.0001 (Table 2, Fig. 2a, b).

**Table 2:** Characteristics for ROC curve analysis of miR-27a and miR-320a

Biomarker	Cutoff	Specificity	Sensitivity
miR-27a	≥ 13.61	100%	100%
miR-320a	≤ 0.122	100%	100%

### Correlation analysis

**Fig. 3:**
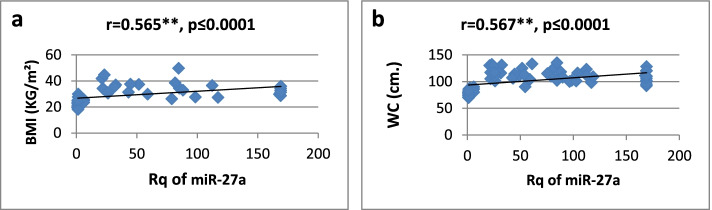
**a**, **b** Correlation coefficient between Rq of MicroRNA 27-a and both BMI (*r* = 0.565) and WC (*r* = 0.567), where “**” means significant at *p* ≤ 0.0001

**Fig. 4:**
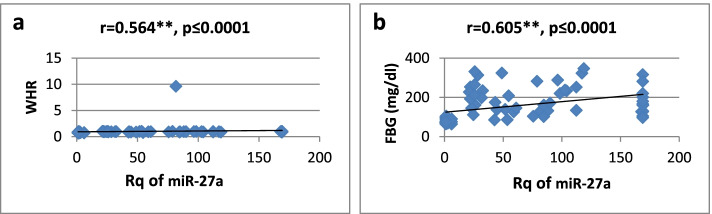
**a**, **b** Correlation coefficient between Rq of MicroRNA 27-a and both WHR (*r* = 0.564) and FBG (*r* = 0.605), where “**” means significant at *p* ≤ 0.0001

**Fig. 5:**
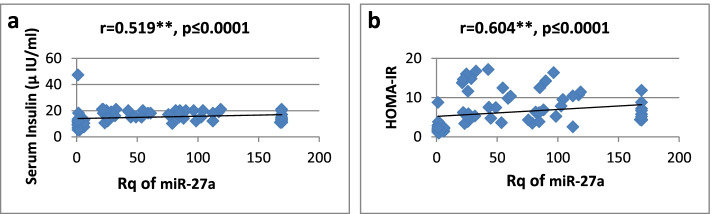
**a**, **b** Correlation coefficient between Rq of MicroRNA 27-a and both serum insulin (*r * = 0.519) and HOMA-IR (*r* = 0.604), where “**” means significant at *p* ≤ 0.0001

**Fig. 6:**
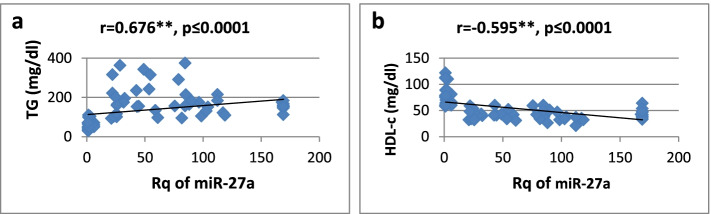
**a**, **b** Correlation coefficient between Rq of MicroRNA 27-a and both TG (*r* = 0.676) and HDLCholesterol (*r* = −0.595), where “**” means significant at *p* ≤ 0.0001

**Fig. 7:**
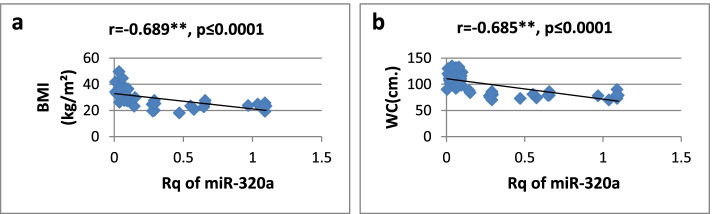
**a**, **b** Correlation coefficient between Rq of MicroRNA 320-a and both BMI (*r* = −0.689) and WC (*r* = −0.685), where “**” means significant at *p* ≤ 0.0001

**Fig. 8:**
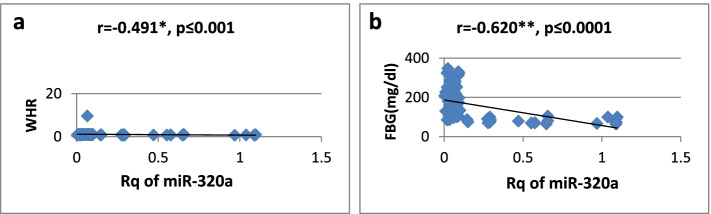
**a**, **b** Correlation coefficient between Rq of MicroRNA 320-a and both WHR (*r* = −0.491), where “*” means significant at *p* ≤ 0.001 and FBG (*r* = −0.620), where “**” means significant at *p* ≤ 0.0001

**Fig. 9:**
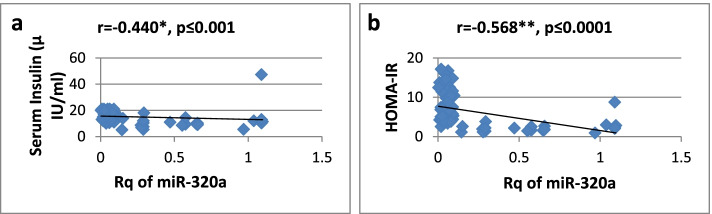
**a**, **b** Correlation coefficient between Rq of microRNA 320-a and both serum insulin (*r* = −0.440), where “*” means significant at *p* ≤ 0.001 and HOMA-IR (*r* = −0.568), where “**” means significant at *p* ≤ 0.0001

**Fig. 10:**
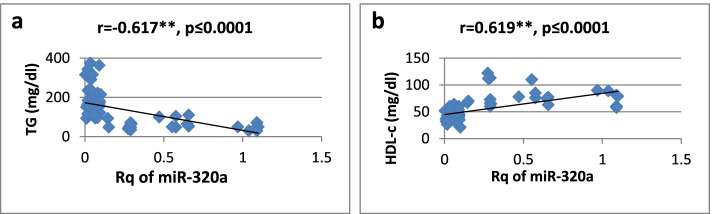
**a**, **b** Correlation coefficient between Rq of microRNA 320-a and both TG (*r* = −0.617) and HDL-cholesterol (*r* = 0.619), where “**” means significant at *p* ≤ 0.0001

As shown in Table 3, there was a high significantly positive correlation between miR-27a expression and BMI, WC, WHR, FBG, insulin, HOMA-IR, and TG, while it showed a significantly negative correlation only with HDL-cholesterol. Regarding relative expression of miR-320a, it showed strong significant negative correlation with BMI, WC, FBG, HOMA-IR, and TG. Serum insulin and WHR showed significantly moderate correlation. MicroRNA 320-a showed significantly positive correlation with HDL-cholesterol. Correlations are represented graphically in Figs. 3, 4, 5, 6, 7, 8, 9, and 10.

**Table 3:** Spearman’s rank correlation coefficient (*r*) of microRNA 27-a and microRNA 320-a with established clinical variables where “*r*” is considered significant at *P* ≤ 0.0001

	***Spearman’s rank correlation coefficient (r) of miR-27a***	***Spearman’s rank correlation coefficient (r) of miR-320a***
**BMI**	0.565**	−0.689**
**WC**	0.567**	−0.685**
**WHR**	0.564**	−0.491*
**FBG**	0.605**	−0.620**
**Serum insulin**	0.519**	−0.440*
**HOMA-IR**	0.604**	−0.568**
**TG**	0.676**	−0.617**
**HDL-C**	−0.595**	0.619**

*means significant at *P* ≤ 0.001

**means significant with *P* ≤ 0.0001

## Discussion

Metabolic syndrome (MetS) is a multifactorial condition consisting of many interrelated biochemical and anthropometric features including elevated visceral adiposity, fasting hyperglycemia, hypertension, and dyslipidemia, with further vascular and immune complications [26].

In the current study, we investigated expression levels of both miR-27a and miR-320a, in lean adult females not having MetS and in a group of obese females having MetS by qRT-PCR aiming to find out if those two specific microRNAs could be used as reproducible and dependable biomarkers for MetS.

According to the diagnostic values of miR-27a and miR-320a that were investigated via the ROC curves and AUCs, the area under the curves (AUC) for miR-27a and miR-320a was 1.000 for both. The cutoff values for mir-27a were ≥ 13.61, while it was ≤ 0.122 for mir-320a, at absolute sensitivity and specificity (100%) for both. According to these high sensitivity and specificity values, mir-27a and mir-320a could be considered as efficient diagnostic biomarkers of MetS.

In agreement with our results, Alvarez et al. [2] and Karolina et al. [16] reported an elevation in the circulating levels of miR-27a in patients having metabolic syndrome and type 2 diabetes, while miR-320a circulating levels were lower in the obese females having metabolic syndrome group when compared to controls in a population of West Virginia [12].

In our results, we identified a highly significant positive association between miR-27a expression and both BMI and WC as indicators of general and central obesity, respectively. Concomitant with our findings, a positive correlation between serum miR-27a levels and BMI had been revealed in adults in a study by Nunez et al. [24]. Based upon these results, the researchers examined miR-27a expression levels in obese and non-obese children, showing a positive correlation between serum miR-27a levels and BMI [34]. These findings could be explained by Lin et al. [20] as their observations indicated that miR-27 regulated genes function through plugging the transcriptional induction of PPARγ and C/EBPα (the two master regulators of adipogenesis) or by stopping preadipocytes from invading the adipogenesis stage. It had been further clarified that binding of miR-27a and miR-27b to the 3′-untranslated region of PPARγ downregulates its expression in 3T3-L1 preadipocytes (mouse embryonic fibroblast-derived cells, used as an adipogenesis model) and inhibits differentiation into adipocyte [23, 34]. In addition, it was speculated that leptin may affect obesity through modulating miR-27a circulating levels, but this mechanism needs more studies to be further explained [32].

Moreover, Yao et al. [34] found that miR-27a is critical for obesity by regulating IR in adipocytes, as well as acting as a repressor of adipocyte differentiation. In our results, the circulating levels of miR-27a positively correlated with fasting plasma glucose and insulin resistance represented as HOMA-IR.

Skeletal muscle is the keystone of glucose metabolism; as the main body site for consumed glucose disposal in healthy subjects, any defect in this function may cause systemic IR. The physiological basis for miR-27a to motivate skeletal muscle insulin resistance has been explained by the alliance of its high serum levels with obesity and the resulting elevation in secreted exosomal miR-27a from adipose tissue that is taken up by skeletal muscle [17]. On focusing to recognize the cellular mechanism of miR-27a that causes insulin resistance in skeletal muscle, the attention was directed towards peroxisome proliferator activated receptor gamma (PPARγ) since miR-27a has been shown as a negative modulator of it **[**27]. PPARγ plays a critical role in skeletal muscle IR by affecting glucose uptake [32].

Moreover, it was found that the altered expression of miR-27a in L6 rat myoblasts prepared in order to be used as an in vitro method reduces glucose consumption and glucose uptake and decreases glucose transporter type 4 (GLUT4) expression [36] which is responsible for regulation of glucose uptake by insulin sensitive tissues, mainly muscle and fat tissue [3].

A large number of studies imply microRNAs as key intermediates in lipid homeostasis regulation process. Our results indicated positive correlation with TG and negative correlation with HDL-cholesterol, but it failed to show any significance with total cholesterol (TCHO) or LDL-cholesterol (LDL-C). In an attempt to understand the relationship between miR-27a and lipid metabolism, Ji et al. [15] clarified that retinoid X receptor alpha (RXRα) has a crucial effect on adipogenesis via making a heterodimer with PPARγ and other nuclear receptors. They added that both miR-27a and miR-27b are illustrated to target RXRα and modulate fat metabolism.

In contrast to our results, Shirasaki et al. [30] investigated the cellular levels of TG and total cholesterol (TCHO) in Huh-7.5 cell line in which miR-27a was inhibited or overexpressed, respectively. TG increased in a dose-dependent manner. Pre-miR-27a suppressed this TG increase, while anti-miR-27a accelerated it. In addition, pre-miR-27a inhibited the increase in TCHO in the culture medium, while anti-miR-27a significantly accelerated it. These conflicting results may be due to the small number of in vivo studies.

Our results showed strong negative significant correlation between miR-320a and both BMI and WC. Concomitant with our results, Munetsuna et al. [22] noticed that miR-320a could be identified as biomarker for obesity. They investigated the circulating microRNAs profile in 526 participants recruited for medical examinations and found that the changed levels of miR-320a expression were significantly correlated with visceral adipose tissue levels, which recommended that miR-320a might be a possible adiposity biomarker. Our study also proved significantly negative correlation of MiR-320a expression with high FBG and HOMA-IR levels.

In the same line, Zampetaki et al. [35] spotted the T2DM-related expression pattern of circulating microRNAs via examining blood samples from over 800 Italian participants selected randomly. This prospective study manifested decreased plasma levels of miR-320 in 80 participants with either diabetes or prediabetes. It was notable that the alteration in miR-320 expression was prior to T2DM diagnosis; hence, the circulating miR-320 could be a suitable predictor for the proceeding T2DM and its resulting health problems.

One possible explanation which could be of particular relevance for understanding the mechanisms underlying our results was in a gene expression analysis made by Ahmed et al. [1] as they found that downregulation of miR-320a caused downregulation of AKT and VEGF genes. AKT is essential for the insulin-mediated effect on metabolism as it starts by the stimulation of glycogen synthesis via Akt-mediated inhibition of glycogen synthase kinase-3, and glycogen synthase [31], while VEGF overexpression leads to an increase in brown adipose tissue (BAT) thermogenesis and also promotes a “BAT-like” phenotype in white adipose tissue depots which may protect against diet-induced obesity and insulin resistance [9].

Conversely, Karolina et al. [16] found an increase in the circulating levels of miR-320 in patients with T2DM and noticed a positive association between the miR-320 level and fasting blood glucose level by analyzing microRNA expression in the blood and exosomes from 265 individuals with distinct health conditions accompanied by MetS. Another research from Singapore identified a positive correlation between miR-320a circulating level and fasting blood glucose levels; overexpression of miR-320a in participants with metabolic syndrome and T2DM was also detected [12].

Our study proved a significant negative correlation between miR-320a relative expression and TG and a significant positive correlation with HDL-cholesterol. In their study, Du et al. [6] revealed that the altered expression of miR-320 might induce lipid and glucose metabolism disturbance in the liver, which may result in metabolic diseases progression.

Contrary to our results, Chen et al. [4] qRT-PCR assays and microarray analysis indicated that the expression of miRNA-320a was upregulated in coronary artery disease patients. Moreover, their in vivo study revealed that overexpression of miR-320a gave rise to a significant increase in plasma lipid levels (TCHO, TG, and LDL).

These controversial findings might be due to many limitations. First, each research is studying the profiling of microRNAs in distinct tissue types which might give rise to specific pathological processes through several mechanisms. Second is their study in proteins and body fluids at divergent pathophysiological disease phases. The third limitation for our study is the small sample size; a larger number of specimens are needed for more explanatory and informative results. Additionally, microRNA panels involved in controlling the related genes and pathways of main MetS components are urgently demanded [14].

## Conclusion

In summary, our research study gives a new insight into the effect of miR-27a and miR-320a on metabolic syndrome different parameters. Although our results demonstrated that both microRNAs showed a significant correlation to the metabolic syndrome in a population of Egyptian females, but thus far, the knowledge about microRNAs biological roles is still partial and incomplete. Moreover, there are many other microRNAs engaged in regulating the related genes and pathways of main metabolic syndrome components. Hence, future studies are much needed in order to help in finding out the potential role of microRNAs as novel biomarkers to predict the people at risk of developing metabolic syndrome, severe health problems, and complications. Additionally, microRNA panels involved in controlling the related genes and pathways of main MetS components are urgently demanded.

## Data Availability

Data sharing is not applicable to this article as no datasets were generated or analyzed during the current study.

## Abbreviations

AKT: Protein kinase B
C/EBPα: CCAAT-enhancer-binding proteins
FBG: Fasting blood glucose
GLUT4: Glucose transporter type 4
HDL: High-density lipoprotein
IR: Insulin resistance
LDL: Low-density lipoprotein
MetS: Metabolic syndrome
NCEP ATP III: National Cholesterol Education Programs Adult Treatment Panel III
ncRNAs: Noncoding RNAs
PPARγ: Peroxisome proliferator-activated receptor gamma
RXRα: Retinoid X receptor-alpha
TG: Triglycerides
VEGF: Vascular endothelial growth factor
WC: Waist circumference
WHR: Waist-hip ratio
